# The Jena Method: Perfusionist Independent, Standby Wet-Primed Extracorporeal Membrane Oxygenation (ECMO) Circuit for Immediate Catheterization Laboratory and/or Hybrid Operating Room Deployment

**DOI:** 10.3390/jcm13051292

**Published:** 2024-02-24

**Authors:** Franz Haertel, Mirko Kaluza, Jurgen Bogoviku, Julian Westphal, Michael Fritzenwanger, Ruediger Pfeifer, Daniel Kretzschmar, Torsten Doenst, Sven Moebius-Winkler, P. Christian Schulze

**Affiliations:** 1 Department of Internal Medicine I, Cardiology, University Hospital Jena, Am Klinikum 1, 07747 Jena, Germany; 2 Department of Cardiothoracic Surgery, University Hospital Jena, Am Klinikum 1, 07747 Jena, Germany; 3 Heart and Vascular Medicine (HUGG), Fleischscharren 4, 38642 Goslar, Germany

**Keywords:** ECMO, ECLS, primed circuit, wet circuit, cardiac resuscitation, low cardiac output support, intensive care, shock, cardiac arrest

## Abstract

**Background:** The timely initiation of extracorporeal membrane oxygenation (ECMO) is crucial for providing life support. However, delays can occur when perfusionists are not readily available. The Jena Method aims to address this issue by offering a wet-primed ECMO system that can be rapidly established without the perfusionist’s presence. **Methods:** The goal was to ensure prompt ECMO initiation while maintaining patient safety. The method focuses on meeting hygienic standards, safe primed storage of the circuit, staff training, and providing clear step-by-step instructions for the ECMO unit. **Results:** Since implementing the Jena Method in 2015, 306 patients received VA-ECMO treatment. Bacterial tests confirmed the sterility of the primed ECMO circuits during a 14-day period. The functionality of all the components of the primed ECMO circuit after 14 days, especially the pump and oxygenator, were thoroughly checked and no malfunction was found to this day. To train staff for independent ECMO initiation, a step-by-step system involves safely bringing the ECMO unit to the intervention site and establishing all connections. This includes powering up, managing recirculation, de-airing the system, and preparing it for cannula connection. A self-developed picture-based guide assists in this process. New staff members learn from colleagues and receive quarterly training sessions by perfusionists. After ECMO deployment, the perfusionist provides a new primed system for a potential next patient. **Conclusions:** Establishing a permanently wet-primed on-demand extracorporeal life support circuit without direct perfusionist support is feasible and safe. The Jena Method enables rapid ECMO deployment and has the potential to be adopted in emergency departments as well.

## 1. Introduction

Extracorporeal life support (ECLS) either due to severe cardiac (veno-arterial extracorporeal membrane oxygenation (VA-ECMO)) or respiratory (veno-venous (VV) ECMO) failure are increasingly being used in catheterization laboratories (CL) and hybrid operating rooms (HOR) [1,2].

However, the successful deployment of ECMO requires preparation and prompt initiation, which can be difficult to achieve in time-sensitive situations such as cardiogenic shock [1,3]. It involves pre-filling the circuit with a sterile solution to minimize the time required for priming and reduce the risk of air embolism during deployment.

In most heart centers, perfusionists are on site and play a crucial role in preparing, resourcing, and starting ECMO units. Unfortunately, they may not always be available in the vicinity of a CL and/or HOR or may be outside of the hospital during on-call duty, which can result in significant delays in establishing life/cardiac output or lung support. This often leads to the need for ECMO units to be transferred, causing further delays. The Jena Method offers a solution by enabling the rapid establishment of the extracorporeal circuit without the need for a perfusionist’s immediate presence, thereby eliminating delays.

Several studies have investigated the efficacy and safety of wet priming in ECMO deployment [4,5,6,7,8,9,10,11]. It was shown that a pre-primed circuit can maintain adequate function for up to two weeks on standby [9]. Another investigation demonstrated the feasibility of using pre-primed circuits in neonatal ECMO, with improved response times and reduced risk of circuit contamination [11]. Furthermore, wet priming has been shown to be an effective method for maintaining the sterility of ECMO circuits for up to 35 days of follow-up [10]. In particular, the Extracorporeal Life Support Organization (ELSO) provides specific recommendations regarding wet primed circuits: “The circuit can be primed at the time of use, or days before. It is not recommended to use a primed circuit after 30 days” [12].

However, the focus of our study is to introduce a new method of wet priming that we call the Jena Method. The Jena Method is designed to not only prepare the ECMO circuit for immediate deployment but also to train the staff on the proper deployment of ECMO in the absence of a perfusionist. With the Jena Method, we hope to improve the accessibility and effectiveness of ECMO deployment, ultimately leading to improved patient outcomes.

### Aim of This Study

The aim of our work is to introduce the Jena Method, a new wet priming technique and context that expedites ECMO deployment and equips staff to deploy ECMO in the absence of a perfusionist.

## 2. Materials and Methods

### 2.1. The Jena Method

Our objective was to eliminate the need for a perfusionist and ECMO unit transfer, without compromising patient safety in the initial phase of VA-ECMO implantation. Additionally, we aimed to ensure that the system met all the hygienic requirements for optimal patient safety if primed and stored to be readily deployable. 

To achieve this, we identified a secure location in the CL/HOR area with easy access to a power socket, where the primed and set up ECMO unit could be placed. We developed a training plan for CL and HOR staff, along with picture-based step-by-step instructions for the ECMO unit, to facilitate staff understanding and proficiency. We also created a systematic plan for the gradual integration of CL staff into ECMO support processes. 

New members of staff learn and train from colleagues of the CL/HOR. Training sessions by the perfusionists take place once a quarter. The current method for transporting the patient to the ICU is that the perfusionist escorts the transport. After arrival at the ICU, the perfusionist checks the system, coordinates the settings of the circuit with the ICU staff, and makes final controls before leaving the ICU. After the use of the ECMO unit, the perfusionist provides a new primed system directly and brings it to the “parking area”. Daily visits to the intensive care unit by the perfusionist is an established practice in our university clinic. This includes, among other things, the system checks and therapy discussions. Furthermore, the perfusionist department operates on an on-call rotation basis and is readily available via phone for any urgent troubleshooting or inquiries.

### 2.2. Patients

This retrospective data collection analyzes selected parameters from patients who were admitted to the cardiac ICU at the University Hospital Jena between 2015 to 2022 and who were treated with VA-ECMO as extracorporeal cardiopulmonary resuscitation (eCPR) because no return of spontaneous circulation (ROSC) was achieved during conventional CPR within at least 20 min in the context of pre- or in-hospital resuscitation or who experienced unstable circulatory condition despite inotropic therapy. 

### 2.3. Patient Data

Various demographic, clinical, and procedural parameters were collected and anonymously registered. The data was gathered from the electronic medical records of the university hospital using two patient data management systems, COPRA (COPRA System GmbH, Berlin, Germany) and SAP (Walldorf, Germany). Also, emergency medical protocols (emergency medical services (EMS) and the emergency room (ER)) were used for confirming the initial working diagnosis.

### 2.4. Bacteriologic Testing

To verify the sterility of the wet prime circuit, we conducted bacterial load studies on three different ECMO systems over a period of 14 days. Fluid samples were collected from randomly selected ECMO circuits and subjected to testing using microscopy, universal culture media, selective media, and matrix-assisted laser desorption/ionization-time of flight (MALDI-TOF). We exchanged experiences with colleagues who conducted their own bacterial load studies.

### 2.5. VA-ECMO

All patients received an ECMO circuit from the manufacturer Getinge^®^ (Rastatt, Germany). The circuit consists of a preassembled, standard tubing set (BE-PLS 2050, Getinge^®^) that includes the Rotaflow RF-32 centrifugal pump (Getinge^®^, Rastatt, Germany), QUADROX^®^ oxygenator (Getinge^®^, Rastatt, Germany), and standard tubing. The system is operated via the Rotaflow console (Getinge^®^, Rastatt, Germany) mounted at the Rotaflow Sprinter Unit with heater unit HU 35 (Getinge^®^, Rastatt, Germany) and manual blender. The circuit will be established via the complete lower body cannulation of femoral vessels using sheath sizes 17–21 Fr (arterial) and 19–25 Fr (venous). Cannulas used are: Bio-Medicus^®^ (Medtronic, Dublin, Ireland) or HLS cannulae set with BIOLINE COATING^®^ (Getinge^®^, Rastatt, Germany). All patients receive an antegrade limp perfusion via an additional 7 Fr bypass canula (CruraSave^®^ Femoral—Perfusion Set, free life medical GmbH^®^, Aachen, Germany). The circuit priming process typically takes approximately 20–25 min and is accomplished by one perfusionist. The approximate cost for all disposable materials for one circuit is estimated to be between 2500 and 3000 EUR.

### 2.6. Statistics

Analyses were made using SPSS Statistics (version 27.0, SPSS Inc., IBM, Armonk, NY, USA). The baseline parameters are presented descriptively, including whole integers and percentages. The data was analyzed by the Kolmogorov-Smirnov test for normal distribution. The variables are expressed as mean ± standard deviation (Mean ± SD).

## 3. Results

In October 2015, the newly built CL/HOR was put in operation, and we started with the establishment of the the Jena Method. Since then, 306 patients received VA-ECMO treatment in our unit. Table 1 provides information on the characteristics of the study population, the major reasons for ECMO treatment, the time of ECMO treatment, the scores (APACHE II and SAPS), and the CPR data. The study population consisted of individuals with a mean age of 60.4 years, and 73% were male. The major reason for ECMO treatment was cardiogenic shock (92%), and ST-elevation myocardial infarction (STEMI) accounted for 44.1% of cases. ECMO treatment was provided at different times of the day, with 49.3% in the afternoon and 17.7% during the night. The mean APACHE II score was 33.5 ± 8.1, and the SAPS was 66.9 ± 17.1. The mean duration of CPR was 37.9 ± 36.6 min, and 58% of patients received CPR in the hospital.

**Figure 1:**
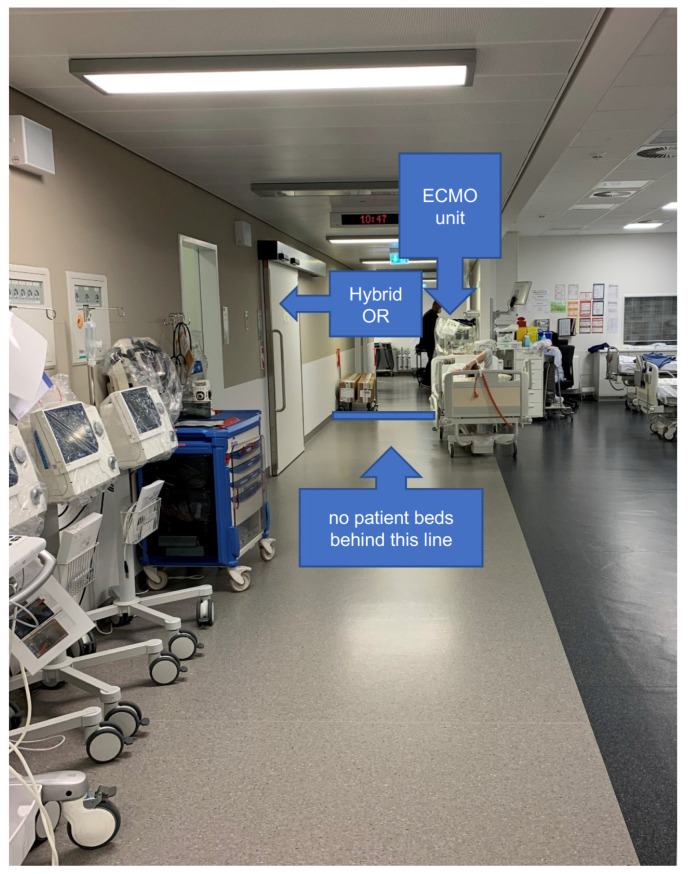
The arrangement of the standby area of the wet-primed ECMO in proximity to the CL and HOR.

In preparation for the opening of the new catherization laboratory unit, the identification of the designated parking area for the ECMO unit was conducted in advance, with diverse requirements. The foremost consideration was the safety of the area, with measures being taken to prevent the damage resulting from inadvertent contact with patient beds, for instance. Additionally, the selected location needed to provide easy access to all CLs and the HOR, as well as having a power socket in close proximity. The resulting parking area is depicted in Figure 1.

Table 2 shows the results of the bacterial growth assessments conducted on three different sets of ECMO systems over a period of 14 days. The assessments were carried out to determine the sterility of the wet prime circuit, and the results indicate that there was no bacterial growth detected in any of the three sets of ECMO systems during the 14-day period.

**Figure 2:**
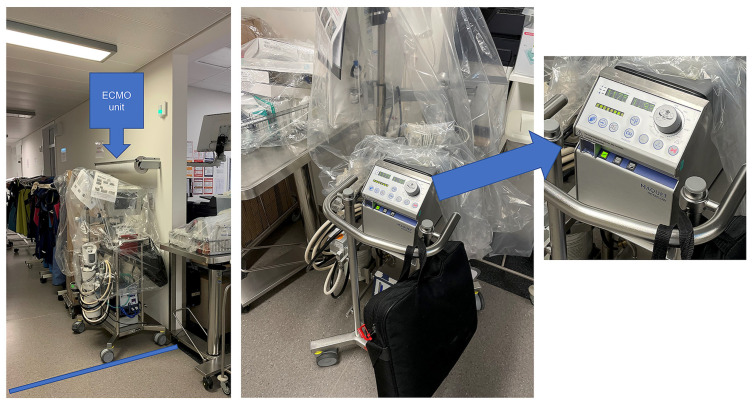
**The** wet-primed ECMO inside the standby area covered (**left**) and in the active closed-loop circulation (**right**) for immediate deployment. The circuit is in active standby, activated (flushed) at least once per week and always prepared on one of the transcatheter aortic valve replacement (TAVR) procedure days.

To shield the circuit, the ECMO unit is covered with a big plastic bag (Figure 2). After 14 days, the primed ECMO circuit and the functionality of all components, especially the pump and oxygenator, were closely monitored and no malfunctions were detected.

**Figure 3:**
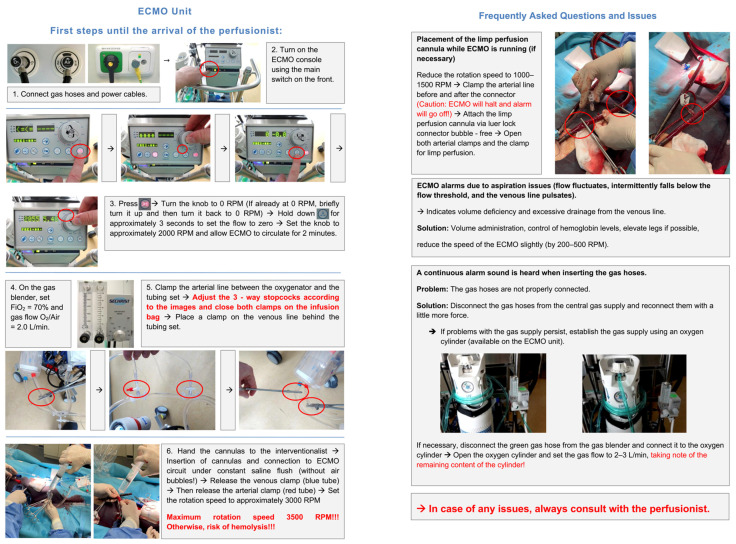
Picture-based step-by-step instruction guide.

The subsequent crucial step, which is repeated consistently, involves acquainting the staff with starting up the console, performing recirculation and deairing of the system after a prolonged period of inactivity, and preparing the system for connection to the cannulas, followed by initiating the system. This training is provided through practical sessions utilizing a training circuit and is reinforced by bed-side instruction when the system is used on actual patients. Subsequently, the CL and HOR staff are empowered to independently start the system with the default settings, aided by a comprehensive, self-developed, picture-based step-by-step instruction manual (Figure 3).

Our data suggests no significant difference in survival rates based on whether the ECMO implantation occurred on weekdays or weekends, or during different times of the day (morning, afternoon, night) during the 5-year period using the Jena Method (Table 3). 

After priming the circuit, the average standby time until deployment was 13.1 ± 5.6 days. Circuits were either deployed directly in the CL or incorporated into a rotation system for use in cardiothoracic surgery procedures within our hospital.

Thus far, no technical complications have arisen concerning the functionality of the circuit itself using the Jena Method.

## 4. Discussion

Since its establishment over seven years ago, the Jena Method has been successfully implemented in CL and HOR, achieving the goal of starting the ECMO/ECLS system without a perfusionist on site in times of staff shortage. The perfusionist department is kept informed when ECMO/ECLS is planned to be implanted, and currently the perfusionist fine-tunes the system and supports patient transport to the ICU. It is being discussed whether these tasks can be taken over by CL staff in the future with proper training and experience. Hospitals without a perfusion department can implement the system with professional training and continuous education. Experienced and well-trained staff in the ICU are required after the implantation of low cardiac output or lung support in CL or HOR. In Europe, the technical aspects of the ECMO program are primarily handled by perfusionists, whereas in the US, this responsibility often falls to ECMO specialists or highly trained nurses. The sterility of the primed circuits is considered hygienically uncritical, and ELSO/Euro ELSO recommendations and literature exist regarding this matter. The clinic recommends discarding the system after 28 days and always uses the freshest system in the CL area. The oldest primed system is used for non-emergency cases, either in the heart surgery department or for ICU implantation, and if enough time is available, it is retrieved from the CL.

The successful implementation of the Jena Method at our CL and HOR has allowed us to start the ECMO/ECLS system without a perfusionist on site, as we aimed to achieve in times of perfusionist shortages. While the perfusionist department is informed whenever ECMO/ECLS is planned to be implanted, they currently perform the fine-tuning of the system and support the transport of the patient to the ICU. However, we are discussing whether these tasks can be taken over by the CL staff in the future. This would require sufficient training to ensure that tasks currently performed by the perfusionist, such as fine-tuning, can be safely performed by the CL staff. With a well-trained and experienced team, this can be accomplished without issues.

Another important consideration is whether the system can be implemented in hospitals without a perfusion department. We believe that this is feasible with proper training and ongoing education. It is worth noting that experienced and well-trained staff in the ICU are also necessary following the implantation of low cardiac output or lung support in the CL or HOR. In Europe, where perfusionists typically take on the technical aspects of the ECMO program, high quality ECMO/ECLS programs have been established. However, it is possible to establish a program without perfusionists, as is currently the case in many ECMO programs for lung support in the ICU [13]. These programs may also involve committed doctors with a higher understanding of the technical aspects of the ECMO program who act as trainers for the staff.

The sterility of the primed circuits is another important consideration in ECMO/ECLS programs. The current recommendations from ELSO/Euro ELSO [12] and the corresponding literature support the fact that primed circuits are hygienically uncritical for defined periods of time [6,8,9,10], and we have not encountered any issues with circuit sterility. However, we recommend discarding the system after a maximum of 28 days. In our clinic, the oldest primed system is always used for non-emergency situations in the heart surgery department or at the implantation in the ICU, and we always use the freshest system in the CL area, unless it is not available.

In summary, the successful implementation of the Jena Method has allowed us to start the ECMO/ECLS system without a perfusionist on site, and we are discussing the possibility of CL staff taking on additional responsibilities in the future. With proper training and education, the system can be implemented in hospitals without a perfusion department. We have not encountered issues with circuit sterility, and we recommend discarding primed systems after a maximum of 28 days. 

Following the implantation, experienced and well-trained ICU staff are essential for daily ECMO functionality assessments and adjustments, overseen by dedicated perfusionists. The research consistently emphasizes the crucial role of highly trained staff in achieving optimal outcomes for VA-ECMO, highlighting perfusionists as the gold standard. It is evident that smaller ECMO centers may yield suboptimal results, underscoring the multifaceted nature of ECMO beyond cannula insertion, necessitating specialized tasks performed by skilled personnel. 

### Limitations

The most noticeable limitation of this study is the absence of a control group from the same time period. All patients were treated using the Jena Method, and there is no comparison with perfusionist involvement against a pre-primed circuit managed by trained staff to validate the circuit’s functionality.

Another potential limitation is that the Jena Method is a transient/temporary solution until a dedicated perfusionist is available. Generally speaking, cannulation could be carried out either by the originating hospital or the receiving hospital [4,14] during transport using the Jena Method, based on the capabilities of each center. However, an established or preexisting ECMO retrieval service is then imperative for providing assistance to patients at institutions lacking the corresponding ECLS capabilities [4,14,15].

The consequential maintenance during the operational phase of the ECMO is still exclusively subject to the perfusionist department. Consequently, making precise assessments regarding the safety of this method is challenging, if absolutely no perfusionists or a perfusionist department would be involved.

The effectiveness of the method appears promising in Jena. However, due to its single-center nature and specialized setup, its generalizability may be limited. Notably, Jena serves as a high-volume ECMO center, having treated over 300 VA-ECMO patients in 7 years. A potential applicability of this method in hospitals with limited perfusionist availability must be discussed and it is crucial for them to address its feasibility in lower-volume centers where outcomes are typically less favorable and VA-ECMO exposure is sporadic.

## 5. Conclusions

We demonstrate the successful implementation of an all-time wet primed ECMO/ECLS circuit standby in the CL and HOR in case of temporary absence of a perfusionist. The Jena Method effectively prevents the delay in establishing the extracorporeal circuit. Our findings suggest that this approach may also be feasible for emergency departments or hospitals that do not have their own perfusionist team. However, this requires access to a perfusionist department at least once every 28 days for ECMO priming. 

Furthermore, the Jena Method can only be a short-term solution/a bridging to transfer a patient to an established ECMO center since well-trained and dedicated staff as well as the necessary resources to maintain the therapy are indispensable. 

It is also conceivable that the Jena-Method could be part of a training method. The availability of a pre-primed circuit may significantly reduce the time required to initiate ECMO/ECLS support in critically ill patients, ultimately improving their outcomes. Further studies are needed to investigate the generalizability of this approach to other healthcare settings and to evaluate its impact on patient outcomes.

## Figures and Tables

**Table 1:** The Jena Method: selected baseline data of treated patients.

	Study Population
	[N = 306]
**Demographics**	
Age [years—mean ± SD]	60.4 ± 14.6
Male [n (%)]	223 (72.9)
Female [n (%)]	83 (27.1)
BMI [kg/m^2^—mean ± SD]	28.8 ± 5.8
Number of comorbidities [mean ± SD]	3.5 ± 2.3
Left ventricular ejection fraction [%—mean ± Std]	24.5 ± 15.5
CAD [n (%)]	214 (69.9)
3-vessel CAD [n (%)]	95 (31)
**Major aetiology for ECMO treatment**	
Cardiogenic Shock [n (%)]	281 (91.8)
STEMI [n (%)]	135 (44.1)
**Time of ECMO treatment**	
Morning [n (%)]	101 (33)
Afternoon [n (%)]	151 (49.3)
Night [n (%)]	54 (17.7)
Weekday admission [n (%)]	235 (76.8)
Weedend admission [n (%)]	71 (23.2)
**Scores**	
APACHE II [mean ± SD]	33.5 ± 8.1
SAPS [mean ± SD]	66.9 ± 17.1
**CPR data**	
Mean duration of CPR [minutes ± SD]	37.9 ± 36.6
In-hospital [n (%)]	178 (58.2)
Out-of-hospital [n (%)]	70 (22.9)
No CPR [n (%)]	58 (18.9)

SD = Standard deviation, STEMI = ST-elevation myocardial infarction, N = absolute number, BMI = Body mass index, CPR = Cardiopulmonary resuscitation, APACHE = Acute Physiology And Chronic Health Evaluation, SAPS = Simplified Acute Physiology Score, CAD = coronary artery disease, ECMO = Extracorporeal membrane oxygenation.

**Table 2:** Results from germ/bacterial growth studies in three wet-primed standby ECMO circuits.

Day after Priming	Bacterial Growth		
	Set 1	Set 2	Set 3
1	none	none	none
2	none	none	none
3	none	none	none
4	none	none	none
5	none	none	none
6	none	none	none
7	none	none	none
8	none	none	none
9	none	none	none
10	none	none	none
11	none	none	none
12	none	none	none
13	none	none	none
14	none	none	none

ECMO = Extracorporeal membrane oxygenation.

**Table 3:** The Jena Method: comparison of survival rates regarding weekdays vs. weekends/regular working hours vs. off-hours after VA-ECMO implantation.

	Survivors	Non-Survivors
	[N = 64]	[N = 242]
**ECMO-implantation**		
Weekdays [n (%)]	50 (78.1)	185 (76.4) *
Weekends [n (%)]	14 (21.9)	57 (23.6) *
Morning [n (%)]	23 (35.9)	78 (32.2) **
Afternoon [n (%)]	32 (50)	119 (49.2) **
Night [n (%)]	9 (14.1)	45 (18.6) **

N = absolute number; ECMO = Extracorporeal membrane oxygenation; * *p* = 0.46 for weekdays vs. weekends; ** *p* = 0.67 for morning vs. afternoon vs. night.

## Data Availability

The data presented in this study are not publicly available due to local legal restrictions on data safety.
